# Breed differences of heritable behaviour traits in cats

**DOI:** 10.1038/s41598-019-44324-x

**Published:** 2019-05-28

**Authors:** Milla Salonen, Katariina Vapalahti, Katriina Tiira, Asko Mäki-Tanila, Hannes Lohi

**Affiliations:** 1 Department of Veterinary Biosciences, University of Helsinki, 00014 Helsinki, Finland; 2 Medicum, Department of Medical Genetics, University of Helsinki, 00014 Helsinki, Finland; 3 The Folkhälsan Institute of Genetics, 00290 Helsinki, Finland; 4 Department of Agricultural Sciences, University of Helsinki, 00014 Helsinki, Finland

**Keywords:** Behavioural ecology, Animal behaviour, Behavioural methods

## Abstract

Cat domestication and selective breeding have resulted in tens of breeds with major morphological differences. These breeds may also show distinctive behaviour differences; which, however, have been poorly studied. To improve the understanding of feline behaviour, we examined whether behavioural differences exist among cat breeds and whether behaviour is heritable. For these aims, we utilized our extensive health and behaviour questionnaire directed to cat owners and collected a survey data of 5726 cats. Firstly, for studying breed differences, we utilized logistic regression models with multiple environmental factors and discovered behaviour differences in 19 breeds and breed groups in ten different behaviour traits. Secondly, the studied cat breeds grouped into four clusters, with the Turkish Van and Angora cats alone forming one of them. These findings indicate that cat breeds have diverged not only morphologically but also behaviourally. Thirdly, we estimated heritability in three breeds and obtained moderate heritability estimates in seven studied traits, varying from 0.4 to 0.53, as well as phenotypic and genetic correlations for several trait pairs. Our results show that it is possible to partition the observed variation in behaviour traits into genetic and environmental components, and that substantial genetic variation exists within breed populations.

## Introduction

The domestication of the cat likely occurred 10 000 years ago in the Near East^1^. In recent years, selection through intentional breeding has resulted in tens of morphologically differing breeds. For example, the International Cat Association currently recognizes 71 breeds^2^. According to cat owners and cat breeders’ organizations, cat breeds also show differences in behaviour. For example, the personality of Ragdoll is described as relaxed, loving, and friendly^3^, whereas Russian Blue cats are described as intelligent, playful, and reserved^4^. These descriptions are usually based on owner and breeder notions and lack scientific basis. However, owners report behaviour differences among cat breeds and non-pedigree house cats^5–7^, and veterinarians recognize behaviour differences at least between the most different breeds^8,9^. As well as showing variation in normal behaviour, cat breeds may differ in susceptibility to abnormal behaviour, as has been observed in dogs^10^. Oriental cats seem to be genetically more susceptible to developing stereotypies than other pedigree and non-pedigree cats^11^. However, research on behaviour differences among cat breeds is sparse and lacks replication, and is focused on few behavioural traits and few breeds. Therefore, more studies are needed to improve the understanding of breed differences in behaviour.

The behaviour of another common companion animal, the dog, has been studied more extensively. Dogs show stable individual^12–15^ and breed differences^16^ in behaviour. Marked breed differences have been discovered in, for example, aggression^17–19^, social and non-social fear^17,19^, playfulness and sociability^20^, boldness^21^, and compulsive behaviour^10,22^. The discovery of such breed differences indicates that behaviour is an inherited feature. Indeed, studies focusing on behaviour and personality have shown considerable level of genetic variation in both humans^23^ and other animals^24^. Dog studies have also found low to moderate^25,26^ heritability in behaviour traits. Furthermore, the estimated genetic correlations among several behaviour traits have also been high^26^.

Cat breeding has historically been based on selecting certain types of cats from locally adapted populations and allowing these favourable types to reproduce. As a result, many cat breeds are still genetically close to the landrace cats they were developed from^27^ and cat breeds from the same region often cluster together in genetic analyses. For example, oriental cat breeds (including Siamese, Burmese and Korat), that were intentionally developed from Asian landrace cats, are genetically distinct from other breeds^27,28^. Similarly, Siberian and Norwegian Forest Cat, that were developed in Northern Europe from local landrace cats, still genetically resemble the random bred populations of their origin^27^. Some cat breeds, however, have a longer breeding history. One of the first established cat breeds was the brachycephalic Persian, with selection for round head and eyes, long hair and a short face starting in the late 19^th^ century^29^. The breed has since been used in the selective breeding of other registered purebred cats, such as Exotic, British Shorthair, Selkirk Rex, and Scottish Fold, and the shared history of these breeds is still seen in genetic clustering^27,28^. The breeds with a shared genetic background may resemble each other not only in morphological traits but also in behaviour. On the other hand, many similar morphological traits have been favoured around the world (for example, long hair, bright coloured eyes, short tail, “blue” coat, and white coat) and convergent evolution may lead to similar behaviour types in genetically distinct cat breeds.

To examine whether cats show breed differences in behaviour, whether cat breeds can be grouped based on their behaviour, and whether cat behaviour is heritable, we used the data of 5726 home-living domestic cats in 40 breeds from our feline health and behaviour questionnaire^30^. For the breed differences, we used logistic regression to compare several behaviour traits in cat breeds. We also included multiple relevant environmental factors in the statistical models, as the living conditions may vary between breeds and affect behaviour. For the grouping of breeds, we used a hierarchical cluster analysis with cat personality traits. For the heritability analyses, we used Bayesian methods on the behaviour data of three breeds with pedigree information and also obtained the genetic and phenotypic correlations between the traits.

## Results

We examined breed differences in behaviour in a sample of 5726 cats in 40 breeds, some of which had to be grouped, forming 19 breeds and breed groups. We used logistic regression and took into account environmental factors (weaning age, access to outdoors, and presence of other cats) as well as general factors (sex and age) by including combinations of these variables in the analyses^31^.

### Breed differences in social behaviour

**Figure 1:**
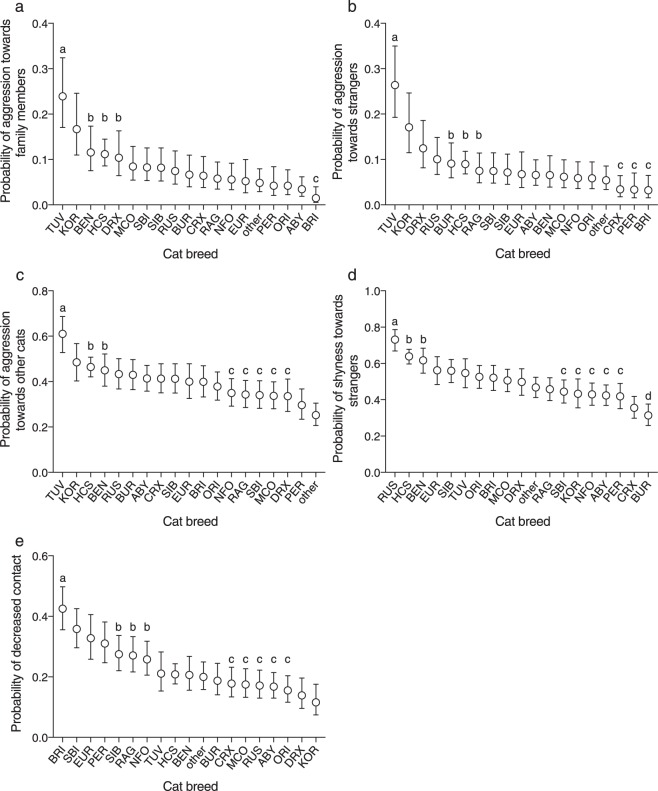
Breed differences in social behaviour in logistic regression analyses. The letterings indicate groups (false discovery rate corrected P > 0.5 between breeds within the same group) that significantly differ (FDR corrected P < 0.05 between breeds in different groups) from other breed groups. Turkish Van cats were the most aggressive towards family members (**a**), strangers (**b**), and other cats (**c**). Russian Blue cats had the highest probability for shyness towards strangers (**d**), and British Shorthair cats had the highest probability for decreased contact to people (**e**). Error bars indicate 95% confidence limits. N = 5726. ABY = Abyssinian, Somali, and Ocicat, BEN = Bengal, BRI = British Shorthair, BUR = Burmese and Burmilla, CRX = Cornish Rex, DRX = Devon Rex, EUR = European Shorthair, HCS = house cat, KOR = Korat, MCO = Maine Coon, NFO = Norwegian Forest Cat, ORI = Balinese, Oriental Longhair, Oriental Shorthair, Seychellois Longhair, Seychellois Shorthair, and Siamese, PER = Persian and Exotic, RAG = Ragdoll, RUS = Russian Blue, SBI = Saint Birman, SIB = Siberian and Neva Masquerade, TUV = Turkish Van and Angora. Odds ratios, their confidence limits, and P-values shown in Supplementary Tables S2–S5 and S7.

The logistic regression analysis detected differences between breeds in social behaviour (Table 1, Fig. 1, Supplementary Tables S2–S5, S7). British Shorthairs had the highest probability for decreased contact to people whereas Korats had the lowest probability. Turkish Vans were the most likely to display aggression towards people. In contrast, British Shorthairs (as well as Persians and Cornish Rex cats in trait ‘aggression to strangers’) had the lowest probability for aggression. Turkish Vans also had the highest probability for aggression towards other cats whereas Persian and ‘other’ cats (consisting of cats belonging to any other breed than shown in Fig. 1) were unlikely aggressive. Russian Blue cats had the highest probability for shyness towards strangers whereas Burmese cats had the lowest probability.

**Table 1:** Association of breed and the response variables in the logistic regression analyses.

Response variable	χ2	P value
Activity level	376.98	**0.0002**
Contact to people	146.68	**0.0005**
Aggression to strangers	117.44	**0.001**
Aggression to family members	110.96	**0.001**
Aggression to cats	104.12	**0.001**
Shyness towards novel objects	171.94	**0.0004**
Shyness towards strangers	196.65	**0.0004**
Wool sucking	103.39	**0.001**
Excessive grooming	43.63	**0.027**
Owner-evaluated behaviour problem	43.24	**0.018**

P values are controlled for false discovery rate. N = 5726 (personality trait analyses), N = 4925 (wool sucking analysis), N = 5683 (excessive grooming analysis), N = 5550 (owner-evaluated behaviour problem analysis). DF = 18. For full results (including the effects of environmental variables), see Table 1 in Ahola *et al*. 2017^31^.

### Breed differences in non-social behaviour

**Figure 2:**
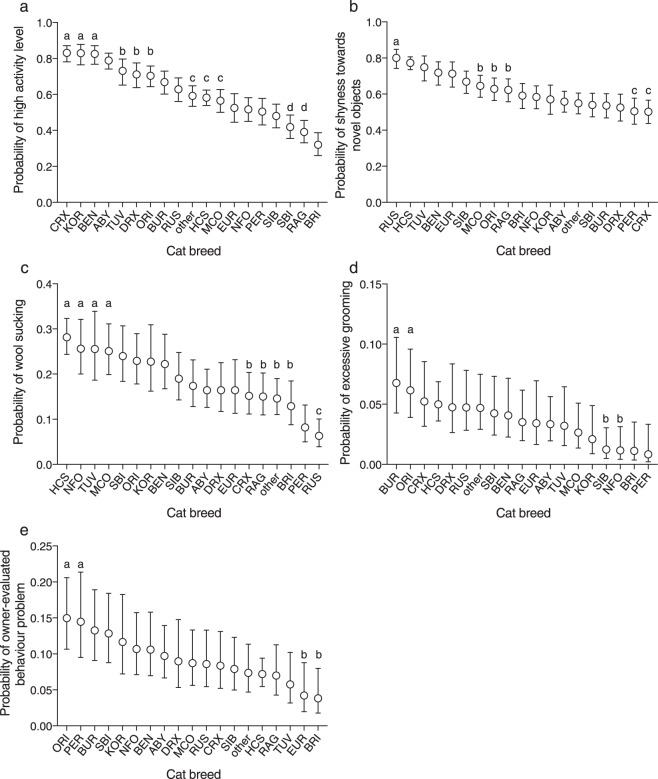
Breed differences in non-social behaviour in logistic regression analyses. The letterings indicate groups (false discovery rate corrected P > 0.5 between breeds within the same group) that significantly differ (FDR corrected P < 0.05 between breeds in different groups) from other breed groups. Cornish Rex cats, Korats, and Bengals had the highest probability for high activity level (**a**). Russian Blue cats had the highest probability for shyness towards strangers (**b**). House cats, Norwegian Forest Cats, Turkish Vans and Maine Coons had the highest probability for wool sucking (**c**), and Burmese and Oriental cats were the most likely to groom excessively (**d**). Oriental and Persian cats were most likely to display an owner-evaluated behaviour problem (**e**). Error bars indicate 95% confidence limits. N = 4925 (wool sucking), N = 5683 (excessive grooming), N = 5550 (owner-evaluated behaviour problem), and N = 5726 (rest). ABY = Abyssinian, Somali, and Ocicat, BEN = Bengal, BRI = British Shorthair, BUR = Burmese and Burmilla, CRX = Cornish Rex, DRX = Devon Rex, EUR = European Shorthair, HCS = house cat, KOR = Korat, MCO = Maine Coon, NFO = Norwegian Forest Cat, ORI = Balinese, Oriental Longhair, Oriental Shorthair, Seychellois Longhair, Seychellois Shorthair, and Siamese, PER = Persian and Exotic, RAG = Ragdoll, RUS = Russian Blue, SBI = Saint Birman, SIB = Siberian and Neva Masquerade, TUV = Turkish Van and Angora. Odds ratios, their confidence limits, and P-values shown in Supplementary Tables S1, S6 and S8–S10.

The logistic regression analysis detected significant differences between breeds in non-social behaviour (Table 1, Fig. 2, Supplementary Tables S1, S6, S8–S10). Cornish Rex, Korat, and Bengal cats were the most active breeds whereas British Shorthairs were the least active. Russian Blue cats were the most likely to show shyness towards novel objects. In contrast, Cornish Rex and Persian cats were the least likely. House cats, Norwegian Forest Cats, Turkish Vans, and Maine Coons were the most likely to perform wool sucking whereas Russian Blue cats were the least likely. Burmese and Oriental cats had the highest probability for excessive grooming. British and Persian cats, in contrast, had the lowest probability. Oriental and Persian cats were the most likely to have an owner-evaluated behaviour problem whereas European and British Shorthairs were the least likely.

### Cluster analysis of breeds by personality traits

**Figure 3:**
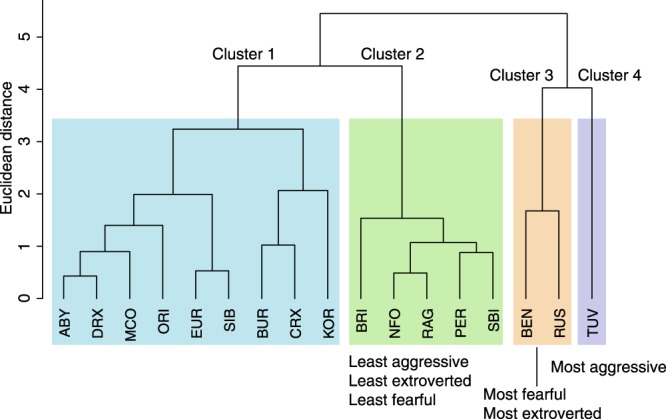
Dendrogram of cat personality traits. Hierarchical cluster analysis grouped cat breeds into four behaviourally distinct clusters. ABY = Abyssinian, Somali, and Ocicat, BEN = Bengal, BRI = British Shorthair, BUR = Burmese and Burmilla, CRX = Cornish Rex, DRX = Devon Rex, EUR = European Shorthair, KOR = Korat, MCO = Maine Coon, NFO = Norwegian Forest Cat, ORI = Balinese, Oriental Longhair, Oriental Shorthair, Seychellois Longhair, Seychellois Shorthair, Siamese, PER = Persian and Exotic, RAG = Ragdoll, RUS = Russian Blue, SBI = Saint Birman, SIB = Siberian and Neva Masquerade, TUV = Turkish Van and Angora.

We performed a principal component analysis to reduce seven behaviour traits (activity level; contact to people; shyness towards strangers and novel objects; aggression towards family members, strangers, and other cats) to personality components. The analysis resulted in three components named aggression, extraversion, and shyness. Based on the personality component scores, cat breeds and breed groups formed four clusters (Fig. 3). When comparing the personality trait means between the clusters, cluster 2 (including British Shorthair, Norwegian Forest Cat, Ragdoll, Persian and Exotic, and Saint Birman) was the least aggressive, extroverted and fearful. Cluster 3 (including Bengal and Russian Blue) was the most fearful and extroverted. Cluster 4 (Turkish Van and Angora) was the most aggressive of all the clusters.

### Heritability and genetic and phenotypic correlations of behaviour

The heritability estimates varied between 0.40 (Ragdoll, shyness towards novel objects and shyness towards strangers) and 0.53 (Ragdoll, aggression to strangers and aggression to family members; Turkish Van, aggression to strangers and aggression to other cats; Table 2). These estimates were quite similar among the breeds.

**Table 2:** Narrow sense heritability of behaviour traits in Ragdoll, Maine Coon, and Turkish Van cats.

	Ragdoll	Maine Coon	Turkish Van
Activity level	0.47 (0.31–0.62)	0.51 (0.37–0.67)	0.51 (0.36–0.68)
Contact to people	0.42 (0.27–0.56)	0.50 (0.35–0.65)	0.50 (0.32–0.68)
Aggression to strangers	0.53 (0.43–0.64)	0.51 (0.41–0.60)	0.53 (0.37–0.68)
Aggression to family members	0.53 (0.45–0.61)	0.48 (0.38–0.59)	0.51 (0.36–0.67)
Aggression to other cats	0.49 (0.33–0.63)	0.46 (0.33–0.59)	0.53 (0.34–0.73)
Shyness towards novel objects	0.40 (0.22–0.58)	0.49 (0.33–0.68)	0.43 (0.23–0.63)
Shyness towards strangers	0.40 (0.22–0.57)	0.47 (0.30–0.67)	0.47 (0.28–0.71)

Highest posterior density interval with 95% probability in parentheses.

**Figure 4:**
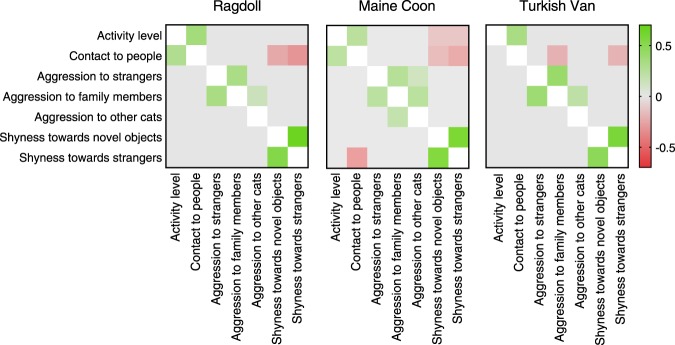
Genetic (below diagonal) and phenotypic (above diagonal) correlation estimates for behaviour traits in Ragdoll, Maine Coon, and Turkish Van cats. Coloured cells indicate correlations that are significantly (HPD interval not including 0) positive (green) or negative (red). The estimates and HDP intervals are shown in Supplementary Tables S11–S13.

Phenotypic correlations were detected in all breeds among extraversion traits, human-directed aggression traits and shyness traits, and between contact to people and shyness towards strangers (Fig. 4, Supplementary Tables S11–S13). We also detected many breed-specific correlations (Fig. 4). The phenotypic correlations varied between 0.66 (Ragdoll, shyness towards strangers and shyness towards novel objects) and −0.31 (Ragdoll, contact to people and shyness towards strangers). All cat breeds showed positive genetic correlations between human-directed aggression traits and between shyness traits (Fig. 4). Furthermore, Ragdolls and Maine Coons showed a positive genetic correlation between contact to people and activity level. Maine Coons also showed a positive genetic correlation between aggression to family members and aggression to other cats, and a negative genetic correlation between contact to people and shyness towards strangers. The genetic correlations varied between 0.61 (Maine Coon, shyness towards strangers and shyness towards novel objects) and −0.35 (Maine Coon, contact to people and shyness towards strangers).

## Discussion

We collected a large data with a survey directed to cat owners and conducted a breed-wise comparison of feline behaviour with over 5700 cats in 19 breeds and breed groups. We studied ten behaviour traits, and, despite including many environmental factors in the statistical analyses, detected breed differences in all traits in both social and non-social behaviour. Furthermore, we clustered the breeds into four behaviourally distinct groups with a cluster analysis. Finally, we discovered that behaviour traits were heritable and many of them were phenotypically and genetically correlated.

Large breed differences were observed in social behaviour. British Shorthair cats had the lowest tendency to seek human contact, whereas Korat and Devon Rex cats were the most likely to seek contact from people. Previously, Persians have been ranked low in friendliness^9^, attention seeking^6^, and sociability^6^, and in our study, they had the highest probability for low contact to people if we only compare breeds that have also been examined in previous studies. However, some previous studies have ranked Abyssinian and Oriental cats low in friendliness^9^, and affection^8,9^, whereas in this study, Abyssinian and Oriental cats were likely to seek contact. In one study, however, Abyssinians showed increased sociability compared to other breeds^7^. Turkish Van and Angora cats were the most aggressive towards both people and other cats. To our knowledge, the behaviour of these breeds has not been previously studied. In previous studies, Bengals^8^ and house cats^5,8^ have been ranked high in aggression (but not in Duffy *et al*. 2018^6^), which was also observed in this study. Similarly, one previous study discovered decreased aggressiveness in Persians^7^, which were among the least aggressive breeds in our study as well. Russian Blue cats had the highest probability for shyness towards strangers, contrasting earlier studies^8,9^. Furthermore, Abyssinians^8,9^ and Persians^8^ were ranked as the most fearful in previous studies, but in our study, these breeds were among the least shy.

Cat breeds differed in non-social behaviour as well. The most active breeds were Cornish Rex, Korat, and Bengal, with British Shorthair, Ragdoll, and Saint Birman being the least active. Previous studies have also examined the activity level of these breeds (not including Cornish Rex) with similar results^6–9^. Russian Blue cats were the shyest towards novel objects. Similarly to shyness towards strangers, this finding is in contrast with previous research^9^. House cats, Norwegian Forest Cats, Turkish Van and Angora cats, and Maine Coons had the highest probability for wool sucking. Burmese and Oriental cats had the highest probability for excessive grooming. Oriental breeds have been suggested to have a higher risk for developing stereotypies^32^ and one recent study also revealed increased compulsive behaviour in Oriental cats^7^. Our finding in stereotypic excessive grooming agrees with these results.

The results of this present study and previous studies^6–9^ are surprisingly consistent, despite that studies have been conducted in different continents and using different methodology. This study, as some previous studies^5–7^, used questionnaires directed to cat owners, whereas two previous studies used ratings by veterinary practitioners^8,9^. None of these approaches has been validated, although questionnaires distributed to owners and carers usually have high predictive validity^33–35^, and thus the next step in the study of cat behaviour would be the validation of methods. The slight differences between the present study and the previous studies may result from genetic divergence of breed populations in different continents, as we focused on Northern European cats whereas previous studies were conducted in USA^6–8^ and Japan^9^.

House cats were, compared to the average purebred cat, moderately active, quite aggressive towards both people and other cats, and shy towards novel objects and strangers. Furthermore, they had a high probability of wool sucking, but owners were not likely to state that the cat has a behaviour problem. House cats of our dataset are Finnish cats from locally adapted populations that have not been under strong artificial selection unlike purebred cats. Based on the questions about the living environment, most adult house cats live in similar conditions as purebred cats in our sample. However, it is likely that the environment in early life differs between the house cats and the purebred cats. It would be expected that pedigree cat breeders invest more time to socialising the kittens. Therefore, some of the behaviour differences between house cats and pedigree cats may be caused by differences in the early environment.

The cluster analysis grouped British Shorthair, Norwegian Forest Cat, Ragdoll, Persian, and Saint Birman as the least aggressive, the least extroverted and the least fearful. Interestingly, British Shorthair breed has been developed from crossbreeding Persians. All of these breeds are also longhaired. Turkish Van cats formed one cluster as the most aggressive breed. This breed was the only Mediterranean basin breed^27^ in our dataset. Bengal and Russian Blue cats formed one cluster, being the most fearful and the most extroverted compared to the other clusters. The personality factor extraversion included both contact to people (sociability) and activity level, and both of these breeds were highly active, explaining this counterintuitive result. This cluster and the cluster containing the majority of the cat breeds (Abyssinian, Devon and Cornish Rex, Maine Coon, Oriental, European Shorthair, Siberian, Burmese, and Korat) consisted mostly of shorthaired cat breeds of European and Asian origin^27^. One previous study^9^ grouped Persian and Ragdoll cats together, similarly to our study, but this group also included Maine Coon cats that clustered differently in our study. Furthermore, this previous study grouped Russian Blue cats with Abyssinian and Siamese cats, unlike our analysis. As the cluster analysis resulted in only four clusters and our data was only moderately clusterable, it is not possible to hypothesize whether the similarity of cat breeds within a cluster result from a shared genetic background or convergent evolution.

The heritability estimates of behaviour varied between 0.40 (shyness) and 0.53 (aggression). This study is, to our knowledge, the first to examine heritability of behaviour in cats, so we cannot compare these estimates to previous findings. In dogs, however, the heritability of behaviour has been estimated in multiple studies and the estimates have varied depending on the studied breed as well as used phenotyping (including, for example, questionnaires and behaviour tests) and statistical methods. Heritability estimates of non-social and stranger-directed fear have varied from 0.25 to 0.36 and from 0.14 to 0.25, respectively^25,36^ and in one study, heritability of nervousness was estimated to be 0.58^37^. The heritability of activity was 0.53 in one study^38^ and the estimates for the heritability of sociability have varied between 0.10 (attachment) and 0.42^25,38^. Owner-directed aggression had a low heritability in two studies^25,36^. The heritability estimates of stranger-directed aggression have varied between 0.24 and 0.26^25,36^ and the estimates of dog-directed aggression between 0.09 and 0.17^25,36^. However, one study conducted with aggressive Labrador retrievers and relatives of aggressive dogs discovered much higher estimates^39^. In conclusion, compared to dog behaviour studies, our heritability estimates fall between the previous estimates.

In our study, genetic correlations varied between −0.32 and 0.63. High genetic correlation estimates were mostly found among traits composing the personality factors, especially between shyness towards strangers and novel objects, and aggression to strangers and owners. Ragdolls and Maine Coons also showed a genetic correlation between activity level and contact to people (both included in the personality factor Extraversion) and Maine Coons showed a negative genetic correlation between shyness towards strangers and contact to people. In previous studies of dog behaviour, fearfulness was highly genetically correlated across contexts^40,41^. Similarly, aggression was shown to correlate across contexts^40,41^, and the genetic correlations between sociability and playfulness as well as between sociability and exploration were also high^41^. In this previous study, dogs also showed a negative genetic correlation between sociability and fearfulness^41^. Furthermore, the genetic correlation estimates of our study are supported by previous studies. We found significant genetic correlations mostly within personality factors, indicating that the traits are both phenotypically and genetically associated. Interestingly, we discovered a positive genetic correlation between contact to people and activity level in Ragdolls and Maine Coons. Ragdoll breed is characterised by its calm and relaxed personality^3^. Thus, when preferring calm and inactive cats in breeding, Ragdoll breeders may unintentionally favour cats seeking little contact to people. Based on our results, the low tendency to seek human contact correlates with low activity level and long hair, whereas in dogs, low levels of attachment is seen in ancient breeds^19^. The genetic correlation estimates of the Ragdoll breed indicate that the low level of contact in these longhaired cats may be a by-product of selection for low activity level to ease handling (especially brushing).

The breed differences in behaviour were detected in our previous study^30^ and these differences persisted even when controlling for many environmental factors. Our results show that as often stated by cat enthusiasts, breeders, and organizations, cat breeds have diverged behaviourally. This finding also indicates that the differences in cat behaviour are inherited, which was confirmed by the heritability analyses in three cat breeds. These analyses showed that all of the behaviour traits studied are moderately or highly heritable and personality factors (extraversion, fearfulness, and aggression) are composed of not only phenotypically, but also genetically correlated traits. Therefore, breeding programs using personality as a main selection criterion could lead to less unwanted behaviour, and thus improve cat welfare. For example, amiability (friendliness) of the cat is associated with higher owner satisfaction, attachment, and quality of bond with the cat, as well as a lower likelihood to find the cat troublesome or problematic^42^. In future, larger studies are required to replicate current results, particularly to improve accuracy of the heritability estimates. We are currently launching a more comprehensive behaviour and personality survey to further evaluate the importance of the living environment and genetic variation in behaviour.

## Methods

### Questionnaire

We designed an extensive online feline health and behaviour questionnaire to collect information on the health, behaviour, and living conditions of Finnish cats. We studied ten behaviour traits: activity level; tendency to seek human contact (labelled as ‘contact to people’); aggressiveness towards human family members, strangers, or other cats; shyness towards strangers or novel stimuli; level of self-grooming (labelled as ‘excessive grooming); wool sucking; and owner-evaluated behaviour problem, which were all coded on a 5-point Likert-type scale^31^, except wool sucking, which was coded 1–8 and behaviour problem, which was coded as ‘no behaviour problem’, ‘self-evaluated behaviour problem’, and ‘behaviour problem diagnosed by a veterinarian’. The questionnaire also included several questions about the background and living environment of the cat, which we utilized in the analyses.

Informed consent was obtained from all participants. Participants agreed that all questionnaire answers could be used for research. We emphasized that all data will be handled strictly confidentially, and that individual cats and owners cannot be recognized from the published results.

### Statistical analyses

Before analyses, we merged some cat breeds together based on known genetic relationships^27,43^, as many breeds had quite small sample sizes. Group ABY included Abyssinian, Ocicat, and Somali. Group BUR included Burmese and Burmilla. Group ORI consisted of Balinese, Oriental Shorthair and Longhair, Seychellois Shorthair and Longhair, and Siamese. Group SIB included Siberian and Neva Masquerade, and group TUV consisted of Turkish Van and Angora. The remaining breeds with small sample sizes (American Curl, American Shorthair, Chartreux, Cymric, Don Sphynx, Kurilian Bobtail, Manx, Egyptian Mau, Sphynx, Selkirk Rex, and option other breed) were combined under ‘other’ breed group. Initially, the data consisted of 7397 cats. After excluding individuals with missing or clearly false responses^31^, the data consisted of 4925 cats in wool sucking, 5683 cats in excessive grooming, 5550 cats in owner-evaluated behaviour problem, and 5726 cats in other traits.

Logistic regression was used to study the breed differences in behaviour in the large dataset. We focused on the ten behaviour traits and used them as response variables in the analyses. They were used as binary traits. In all aggression and shyness traits, the event constituted of levels 2–5 on the 5- point Likert scale. In grooming, the event was 4–5. In contact with people, a low level of contact (between 1–3) constituted the event. In wool sucking, levels 4–8 (at least monthly) constituted the event and level 1 (never) the non-event. In behaviour problem, the self-diagnoses and veterinary diagnoses were grouped together and constituted the event. As we were interested in breed differences that could reflect genetic differences rather than differences in, for example, the living environment, we included several explanatory variables in the analyses in addition to the breed. We used a forward stepwise AIC (Akaike Information Criterion) selection approach to select the models with the best fit. The final models and AIC model selection are shown in Supplementary Table S3 of our previous study^31^.

As the number of pairwise comparisons was high due to a large number of categorical variables, all P values of the logistic regression analyses were corrected for false discovery rate (FDR) to decrease the probability of type I error. The significance cut-off P-value was set at P < 0.05. Furthermore, as it is difficult to show pairwise comparisons between 20 breeds, the breeds were grouped using the false discovery rate corrected P-values. These breed groups consist of breeds that are similar with each other (FDR corrected P > 0.5) and significantly different (FDR corrected P < 0.05) from breeds in other groups. These groups are indicated by letterings in Figs 1 and 2 in the Results section.

For the cluster analysis, we first reduced the behaviour traits into personality components by a principal component analysis with polychoric correlations. Three personality components were extracted and we named these extraversion (activity level and contact to people), aggression (aggression to cats, strangers, and family members), and shyness (shyness towards strangers and novel objects)^31^. We performed an agglomerative hierarchical cluster analysis using these three personality traits. First, we excluded house cats and the breed group ‘other’. Secondly, we calculated the mean trait score for each breed, separately for males and females. As the scores of females and males were highly correlated within breeds, we averaged their scores. Thirdly, we assessed the clustering tendency of the data with the package factoextra^44^ in R. The clustering tendency was measured with the Hopkins statistic, which indicated only moderate clustering (H = 0.375). Finally, the agglomerative hierarchical clustering was performed on the personality components and the results were visualised using package cluster^45^ in R. We used Ward’s minimum variance method as it obtained the highest agglomerative coefficient compared to other clustering criterions. We grouped the breeds into four clusters, as suggested by the majority of indices in package NbClust^46^ in R. After the clustering analysis, we calculated the average personality component scores for each cluster to assess their behavioural differences.

We estimated the (narrow sense) heritability (h^2^) and the genetic and phenotypic correlations of the Likert scale behaviour traits in three cat breeds with relatively large sample sizes: Ragdoll (N = 357), Maine Coon (N = 356), and Turkish Van (N = 157) using Bayesian analysis with a flat prior. The computing was performed with the package MCMCglmm^47^ in R. In the analysis, the heritability estimate is the mean of the posterior distribution, and the highest probability density (HPD) interval for 95% probability is analogous to the conventional confidence interval. In the analyses, we included only cats that were over 6 months old. We used a multivariate model to simultaneously estimate the heritability of the seven behaviour traits that form the personality traits explained above: activity level; contact with people; aggression to family members, strangers, and other cats; and shyness towards novel objects and strangers. We included age (6 months to 17 years) as linear and quadratic covariate, and sex (male and female) and owner identity (60–188 per breed) as categorical fixed effects. Owner identity was defined as ‘other’ for the owners having only one cat in the data. We specified the flat prior with inverse-gamma distribution and ran the analyses with other priors to verify that the prior distribution did not affect the posterior distribution. We ran the models for 10000 iterations, discarded first 1000 iterations and sampled every 10 iterations thereafter, to reach an effective sample size of at least 100. The influence of the individual’s additive genetic effect in the model was tested by removing it from the model and comparing the deviance information criterion (DIC) between the models. In all three breeds, the inclusion of the additive genetic effect decreased model DIC value by over 180 units, indicating substantial support for the effect.

## Supplementary information

Supplementary Tables S1-S10

Supplementary Tables S11-S13

## Data Availability

The anonymized data is available in Figshare: 10.6084/m9.figshare.8143835.
