# Supplementary material for: Breed differences of heritable behaviour traits in cats
Source: Sci Rep. 2019 May 28;9:7949. doi: 10.1038/s41598-019-44324-x (PMC6538663; doi:10.1038/s41598-019-44324-x)
Supplement: Supplementary file 2 — Supplementary Tables S11-S13 [file 41598_2019_44324_MOESM2_ESM.pdf]

Breed differences of heritable behaviour traits in cats

Milla Salonen, Katariina Vapalahti, Katriina Tiira, Asko Mäki-Tanila, Hannes Lohi

Supplementary Table S11. Phenotypic and genetic correlations in Maine Coon.

	Activity level	Contact to people	Aggression to strangers	Aggression to family members	Aggression to other cats	Shyness towards novel objects	Shyness towards strangers
Activity level		**0.29 (0.18, 0.40)**	0.11 (-0.00, 0.24)	0.10 (-0.01, 0.22)	0.07 (-0.06, 0.19)	**-0.17 (-0.28, -0.05)**	**-0.17 (-0.28, -0.04)**
Contact to people	**0.28 (0.05, 0.51)**		0.03 (-0.08, 0.16)	-0.04 (-0.17, 0.07)	-0.01 (-0.13, 0.11)	**-0.21 (-0.31, -0.07)**	**-0.29 (-0.42, -0.18)**
Aggression to strangers	0.14 (-0.06, 0.34)	0.04 (-0.17, 0.25)		**0.32 (0.21, 0.42)**	**0.17 (0.05, 0.29)**	-0.09 (-0.21, 0.05)	-0.10 (-0.22, 0.03)
Aggression to family members	0.10 (-0.11, 0.32)	-0.06 (-0.27, 0.15)	**0.29 (0.11, 0.46)**		**0.29 (0.18, 0.40)**	-0.02 (-0.16, 0.10)	-0.04 (-0.17, 0.08)
Aggression to other cats	0.07 (-0.21, 0.31)	-0.05 (-0.29, 0.19)	0.11 (-0.09, 0.36)	**0.23 (0.03, 0.44)**		-0.02 (-0.10, 0.07)	0.04 (-0.08, 0.15)
Shyness towards novel objects	-0.18 (-0.43, 0.09)	-0.28 (-0.54, 0.01)	-0.10 (-0.32, 0.12)	-0.01 (-0.26, 0.23)	0.14 (-0.13, 0.41)		**0.64 (0.56, 0.70)**
Shyness towards strangers	-0.16 (-0.43, 0.11)	**-0.35 (-0.61, -0.09)**	-0.09 (-0.31, 0.13)	-0.01 (-0.23, 0.26)	0.08 (-0.23, 0.34)	**0.61 (0.43, 0.79)**	

Phenotypic correlations in the upper diagonal, and genetic correlations in the lower diagonal. HPD intervals in the parentheses.

Supplementary Table S12. Phenotypic and genetic correlations in Ragdoll.

	Activity level	Contact to people	Aggression to strangers	Aggression to family members	Aggression to other cats	Shyness towards novel objects	Shyness towards strangers
Activity level		**0.36 (0.25, 0.45)**	0.03 (-0.08, 0.15)	0.05 (-0.07, 0.17)	-0.01 (-0.13, 0.11)	-0.09 (-0.21, 0.03)	-0.04 (-0.15, 0.07)
Contact to people	**0.29 (0.05, 0.57)**		-0.04 (-0.17, 0.07)	-0.04 (-0.16, 0.07)	0.04 (-0.09, 0.15)	**-0.24 (-0.35, -0.13)**	**-0.31 (-0.42, -0.21)**
Aggression to strangers	0.06 (-0.16, 0.25)	-0.02 (-0.25, 0.18)		**0.32 (0.23, 0.43)**	0.11 (-0.01, 0.23)	0.04 (-0.05, 0.16)	0.07 (-0.05, 0.18)
Aggression to family members	0.07 (-0.11, 0.26)	-0.03 (-0.23, 0.15)	**0.33 (0.19, 0.48)**		**0.14 (0.03, 0.26)**	0.02 (-0.09, 0.13)	0.04 (-0.07, 0.16)
Aggression to other cats	0.05 (-0.26, 0.33)	0.09 (-0.17, 0.40)	0.12 (-0.11, 0.32)	0.14 (-0.04, 0.34)		0.01 (-0.04, 0.06)	-0.01 (-0.14, 0.09)
Shyness towards novel objects	-0.01 (-0.33, 0-28)	-0.17 (-0.46, 0.14)	0.04 (-0.16, 0.28)	0.03 (-0.16, 0.23)	0.14 (-0.13, 0.42)		**0.66 (0.59, 0.73)**
Shyness towards strangers	0.02 (-0.32, 0.33)	-0.28 (-0.58, 0.01)	0.08 (-0.14, 0.30)	0.06 (-0.13, 0.28)	0.02 (-0.28, 0.30)	**0.53 (0.29, 0.76)**	

Phenotypic correlations in the upper diagonal, and genetic correlations in the lower diagonal. HPD intervals in the parentheses.

Supplementary Table S13. Phenotypic and genetic correlations in Turkish Van.

	Activity level	Contact to people	Aggression to strangers	Aggression to family members	Aggression to other cats	Shyness towards novel objects	Shyness towards strangers
Activity level		**0.33 (0.16, 0.49)**	-0.09 (-0.27, 0.09)	-0.01 (-0.20, 0.19)	0.03 (-0.16, 0.23)	0.05 (-0.13, 0.23)	0.03 (-0.17, 0.21)
Contact to people	0.28 (-0.06, 0.57)		-0.12 (-0.29, 0.07)	**-0.21 (-0.39, -0.05)**	-0.01 (-0.21, 0.17)	-0.12 (-0.30, 0.06)	**-0.20 (-0.39, -0.03)**
Aggression to strangers	-0.10 (-0.44, 0.22)	-0.11 (-0.47, 0.27)		**0.41 (0.26, 0.57)**	0.06 (-0.12, 0.27)	0.06 (-0.13, 0.24)	0.09 (-0.11, 0.27)
Aggression to family members	-0.03 (-0.37, 0.28)	-0.20 (-0.55, 0.10)	**0.38 (0.13, 0.64)**		**0.21 (0.04, 0.39)**	0.13 (-0.05, 0.34)	0.13 (-0.05, 0.32)
Aggression to other cats	-0.09 (-0.46, 0.25)	-0.08 (-0.44, 0.33)	0.08 (-0.26, 0.41)	0.18 (-0.13, 0.52)		0.09 (-0.04, 0.25)	-0.04 (-0.22, 0.15)
Shyness towards novel objects	0.02 (-0.40, 0.33)	-0.10 (-0.49, 0.27)	0.01 (-0.35, 0.39)	0.06 (-0.32, 0.40)	0.06 (-0.35, 0.45)		**0.58 (0.47, 0.70)**
Shyness towards strangers	0.02 (-0.32, 0.39)	-0.13 (-0.49, 0.25)	0.02 (-0.35, 0.38)	0.05 (-0.29, 0.42)	-0.04 (-0.45, 0.34)	**0.47 (0.16, 0.74)**	

Phenotypic correlations in the upper diagonal, and genetic correlations in the lower diagonal. HPD intervals in the parentheses.
